# Regulation of metabolic gene clusters in *Arabidopsis thaliana*

**DOI:** 10.1111/nph.13189

**Published:** 2014-11-21

**Authors:** Hans-Wilhelm Nützmann, Anne Osbourn

**Affiliations:** 1 Department of Metabolic Biology, John Innes Centre, Norwich Research ParkNorwich, NR4 7UH, UK

**Keywords:** *Arabidopsis thaliana*, ARP6, H2A.Z, nucleosome positioning, plant metabolic gene clusters, specialized metabolism, triterpenes

## Abstract

- Recent discoveries have revealed that the genes for the biosynthesis of a variety of plant specialized metabolites are organized in operon-like clusters within plant genomes. Here we identify a regulatory process that is required for normal expression of metabolic gene clusters in *Arabidopsis thaliana*.
- Comparative gene expression analysis of a representative clustered gene was performed in a set of chromatin mutant lines. Subsequently, metabolite levels were analysed by GC-MS and the local chromatin structure was investigated by chromatin immunoprecipitation and nucleosome positioning.
- We show that the transcript levels of genes within two metabolic clusters are coordinately reduced in an *arp6* and *h2a.z* background. We demonstrate that H2A.Z enrichment in the clusters is positively correlated with active cluster expression. We further show that nucleosome stability within the cluster regions is higher in the *arp6* background compared with the wild-type.
- These results implicate ARP6 and H2A.Z in the regulation of metabolic clusters in *Arabidopsis thaliana* through localized chromatin modifications that enable the coordinate expression of groups of contiguous genes. These findings shed light on the complex process of cluster regulation, an area that could in the future open up new opportunities for the discovery and manipulation of specialized metabolic pathways in plants.

See also the Commentary by Fernie and Tohge

## Introduction

In eukaryotes the arrangement of the genes along the chromosome is not as random as was first thought, and distinctive clusters of functionally related but nonhomologous genes can be found in the genomes of certain animals and fungi (Hurst *et al*., 2004; Michalak, 2008). These include the major histocompatibility complex in mammals and gene clusters for the synthesis of specialized metabolites (such as penicillin and toxins) in filamentous fungi (Osbourn & Field, 2009). Genes for specialized metabolic pathways in plants have until recently been assumed to be dispersed throughout plant genomes, and certainly there are well-known examples of pathways for which this is true (e.g. the phenylpropanoid and glucosinolate pathways). However, over 20 examples of biosynthetic gene clusters for the synthesis of specialized metabolites have now been reported from diverse plant species (Boycheva *et al*., 2014; Nützmann & Osbourn, 2014). These clusters span regions of *c*. 35–270 kb and consist of 3 to 10 genes. They include clusters for the synthesis of hydroxamic acids in maize; terpenes in *Arabidopsis thaliana*, castor, rice and oats; cyanogenic glycosides in *Lotus japonicus*, sorghum and cassava; and alkaloids in poppy, tomato and potato (Frey *et al*., 1997; Qi *et al*., 2004; Wilderman *et al*., 2004; Shimura *et al*., 2007; Field & Osbourn, 2008; Field *et al*., 2011; Nützmann & Osbourn, 2014; Takos *et al*., 2011; Winzer *et al*., 2012; Itkin *et al*., 2013; Krokida *et al*., 2013; Matsuba *et al*., 2013; King *et al*., 2014). The natural functions of these specialized metabolites are likely to be in defence against herbivores and pathogens. Some of these compounds also have major pharmaceutical and agricultural importance, for example, noscapine (an antitumor alkaloid from poppy), and the anti-nutritional steroidal glycoalkaloids found in tomato and potato. Although plant specialized metabolic gene clusters share some features in common with bacterial operons (physical clustering of genes and coordinate regulation of expression) there is a compelling body of evidence to indicate that they have arisen *de novo* by genome reorganization, and that they are not a consequence of horizontal gene transfer from microbes (Field & Osbourn, 2008; Field *et al*., 2011). The regulatory mechanisms that govern coordinate expression of metabolic gene clusters in plants are, for the most part, unknown. So far only one transcriptional regulator has been identified for clustered pathway genes (for diterpene biosynthesis in rice) and it is not clear whether this is pathway specific or whether it has a broader role in regulation of metabolism in rice (Miyamoto *et al*., 2014).

Physical clustering has the potential to enable coordinate regulation of the genes within plant metabolic gene clusters at the level of chromatin. In filamentous fungi there is evidence that metabolic gene clusters are controlled by chromatin-based mechanisms involving histone acetyltransferases, deacetylases, and methyl transferases (Bok *et al*., 2009; Reyes-Dominguez *et al*., 2010; Nützmann *et al*., 2011; Brakhage, 2013). In yeast (*Saccharomyces cerevisiae*) the histone 2 variant H2A.Z is required for the coordinate regulation of the *DAL* cluster, a catabolic gene cluster for allantoin utilization (Wong & Wolfe, 2005). H2A.Z is also involved in the regulation of adaptive gene clusters in other organisms, including the virulence (*vir*) cluster in the malaria parasite (*Plasmodium falciparum*) and the *Hox* gene cluster in animals (Meneghini *et al*., 2003; Creyghton *et al*., 2008; Petter *et al*., 2013).

Here we demonstrate that the histone variant H2A.Z is essential for normal expression of metabolic gene clusters and regulation of metabolite synthesis in *Arabidopsis thaliana*. We show by mutation that inability to incorporate H2A.Z into nucleosomes leads to reduced cluster expression. We show by chromatin immunoprecipitation (ChIP) analysis that activation of clusters is accompanied by increased H2A.Z deposition into nucleosomes in the genic and intergenic regions within the clusters and, by measurement of nucleosome occupancy, with loosening of the local nucleosome structure. Thus we propose that H2A.Z-mediated chromatin modification leads to localized opening of the nucleosome structure and facilitates expression of gene clusters for the synthesis of specialized metabolites in plants.

## Materials and Methods

### Plant material

Unless otherwise stated all plants used in this study were in the *Arabidopsis thaliana* Columbia (Col-0) background. The *arp6* (Col-0) and *vin3-8* lines were provided by Ali Pendle (John Innes Centre, Norwich, UK) and Jean Finnegan (CSIRO Plant Industry, Clayton South, Australia). The *arp6* (L*er*), *hta9/hta11* and *hta11-GFP* lines were donated by Vinod Kumar (John Innes Centre). *Haf1* (SALK_070585C), *hda18* (SALK_006938), *ref6* (SALK_001018C), *vim1* (SALK_050903C), *atjmj4* (SALK_135712C), *elf6* (SALK_074694C), *hda6* (CS66154), *otld1* (SALK_037047C), *atx1* (SALK_149002C), *atxr7* (SALK_149692C), *suvh2* (SALK_079574C), *prmt10* (SALK_047046C), and *prmt5* (SALK_065814C) lines were obtained from the Nottingham Arabidopsis Stock Centre (Scholl *et al*., 2000) and homozygous mutants isolated. Further information about these lines is provided in Supporting Information Table S1. Seedlings were grown in Petri dishes on Murashige and Skoog medium containing 1% sucrose (screening of chromatin mutant lines) and Murashige and Skoog plant salt medium supplemented with 0.5% phytagel and 0.75% sucrose (all other experiments) (Murashige & Skoog, 1962). Petri dishes were maintained vertically in growth chambers (22°C, 16 h light) for 6–7 d.

### Gene transcript level analysis

Total RNA was isolated from roots of seedlings with the RNeasy Plant Mini Kit (Qiagen, Venlo, The Netherlands) and DNA degradation performed with the TURBO DNA-free kit (Life Technologies, Carlsbad, CA, USA). cDNA was prepared using the RevertAid First Strand cDNA Synthesis Kit (Thermo Scientific, Waltham, MA, USA) and quantitative reverse transcription polymerase chain reaction (qRT-PCR) was performed with the MyTaq HS Mix 2x (Bioline, London, UK) in combination with Evagreen (Biotium, Hayward, CA, USA) on a CFX96 Real-Time PCR detection system (Bio-Rad, Hercules, CA, USA). The cycling conditions used were: 95°C for 2 min, followed by 45 cycles of DNA denaturation at 95°C for 5 s and primer annealing and extension at 62°C for 35 s. All qRT-PCR experiments were performed with at least three biological replicates and two technical replicates. Data were analysed with the Bio-Rad CFX manager 3.0. Quantification results were normalized against *PP2AA3* (*At1g13320*) (Hong *et al*., 2010).

### Chromatin immunoprecipitation

ChIP assays were carried out as described previously (Song *et al*., 2014). Sonicated DNA fragments showed average length of 400 bp. Precipitation was performed against anti-GFP antibody (Abcam AB290). All ChIP experiments were quantified by qRT-PCR. H2A.Z abundance at *FLOWERING LOCUS C* (*FLC*) was used as an internal control. Data are shown as the ratio of (H2A.Z-GFP locus of interest/input DNA locus of interest) to (H2A.Z-GFP *FLC*/input DNA *FLC*). Each ChIP experiment was carried out with at least three biological replicates and qRT-PCR quantification was performed in duplicate.

### Nucleosome positioning

Nucleosome positioning was performed as described previously (Kumar & Wigge, 2010). Each positioned nucleosome was determined individually by qRT-PCR using tiled oligos positioned around the putative transcriptional start sites of target genes and in the intergenic region of the gene cluster (Supporting Information Table S2). Stably positioned nucleosomes in intergenic regions were pre-selected based on genome-wide nucleosome positioning data and individually confirmed by qRT-PCR using tiled oligos (Zhang *et al*., 2007). The −1 nucleosome at *At4g07700* was used as an internal control (Kumar & Wigge, 2010). Data are represented as the ΔΔCt value of the region of interest normalized against the −1 nucleosome at *At4g07700* and uncut input DNA. The qRT-PCR cycling conditions were adapted to 54°C annealing temperature for 10 s.

### Metabolite extraction and quantification

Root tissue (*c*. 100 mg) from 6 d old seedlings of wild-type and mutant lines was harvested. Extraction was carried out as described (Field & Osbourn, 2008). The internal standard coprostanol (Sigma Aldrich) was incorporated during saponification. GC-MS analysis was performed as previously described (Field & Osbourn, 2008). For quantification, an extracted ion chromatogram was obtained and thalianol (*m*/*z* 229), coprostanol (*m*/*z* 370) and sitosterol (*m*/*z* 357) peaks were integrated with the RTE integrator (Agilent Technologies, Santa Clara, CA, USA).

## Results and Discussion

**Fig 1:**
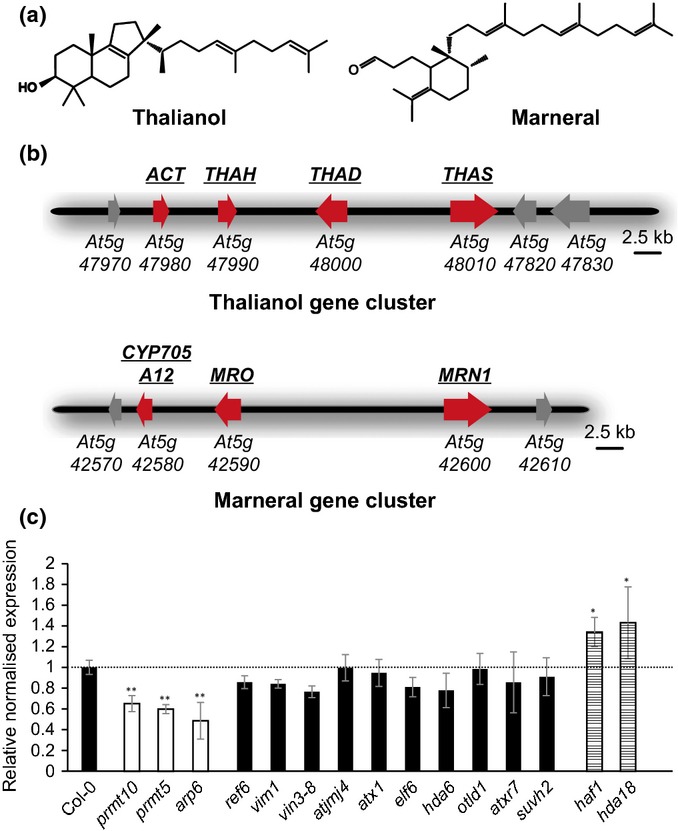
Steady state transcript levels of *THAH*, a representative thalianol cluster gene, in different *Arabidopsis thaliana* chromatin mutant lines. (a) The structures of thalianol and marneral. (b) The thalianol and marneral gene clusters (red, cluster genes; grey, flanking genes). The thalianol cluster consists of an oxidosqualene cyclase gene (thalianol synthase, *THAS*), two nonhomologous cytochrome P450 genes (thalianol hydroxylase, *THAD*; thalian-diol desaturase, *THAH*) and an acyltransferase gene (*ACT*); the marneral cluster consists of one oxidosqualene cyclase gene (marneral synthase, *MRN1*) and two nonhomologous cytochrome P450 genes (*CYP705A12*; marneral oxidase, *MRO*). (c) The relative transcript levels of the thalianol cluster gene *THAH* was measured in roots of seedlings of the Col-0 wild-type and chromatin mutant lines (see Supporting Information Table S1). Gene expression was measured by qRT-PCR and the wild-type transcription level set as 1. *PP2AA3* (*At1g13320*) was used as an internal control (Hong *et al*., 2010). Error bars indicate standard deviation for three biological replicates. Statistical significance (*t*-test): *P <* *0.05; **P <* *0.01.

Previously we have characterized two gene clusters for the synthesis of specialized metabolites in *Arabidopsis thaliana*, the thalianol cluster and the marneral cluster (Field & Osbourn, 2008; Field *et al*., 2011). The thalianol and marneral clusters have evolved independently and encode enzymes for the synthesis and elaboration of the triterpenes thalianol and marneral, respectively (Fig.1a,b). These pathways have both been implicated in development and in ecological interactions (Field & Osbourn, 2008; Field *et al*., 2011; Go *et al*., 2012). The genes within each of these two clusters are co-regulated and are expressed exclusively in roots (Supporting Information Fig. S1) (Field & Osbourn, 2008; Field *et al*., 2011).

To assess the potential role of chromatin in regulation of plant metabolic gene clusters we first evaluated the transcript levels of a representative gene from one of these clusters, the thalianol cluster (the gene encoding thalianol hydroxylase (THAH)) in a set of *Arabidopsis thaliana* mutants affected in histone modifications and chromatin remodelling (Supporting Information Table S1). *THAH* transcript levels were clearly reduced in three of these lines relative to the Col-0 wild-type (the histone methyltransferase mutants *prmt5* and *prmt10*; and *arp6*, which is mutated in a gene encoding a component of a chromatin remodelling complex) and elevated in two (the histone acetyltransferase mutant *haf1* and the histone deacetylase mutant *hda18*) (Fig.1c). The *arp6* mutant line had the most pronounced negative effect on *THAH* gene transcript levels of all the mutant lines investigated. ARP6 is part of the SWR1 chromatin remodelling complex and is required for the deposition of the histone variant H2A.Z into nucleosomes (Deal *et al*., 2007; Kumar & Wigge, 2010). The potential involvement of H2A.Z in regulation of plant metabolic gene clusters raises intriguing parallels with the *DAL* gene cluster in yeast, where H2A.Z deposition is associated with activation of cluster gene expression (Meneghini *et al*., 2003; Wong & Wolfe, 2005). We therefore focused our subsequent investigations on ARP6.

**Fig 2:**
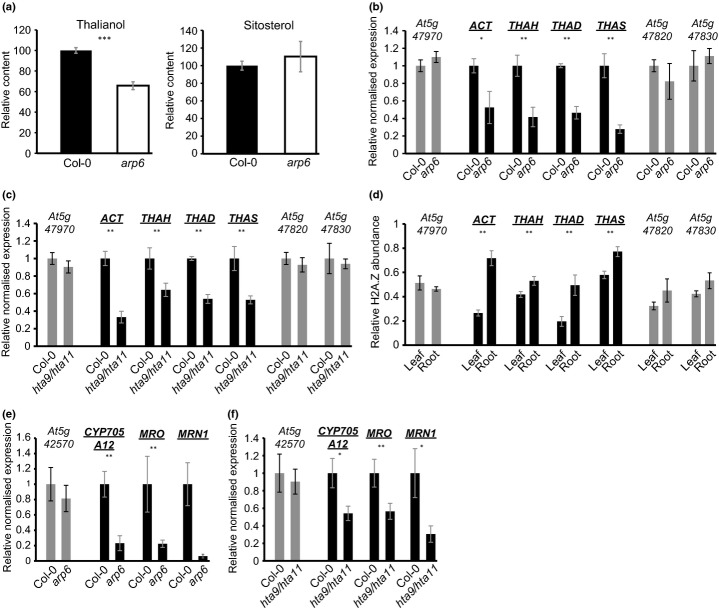
Mutation of the *ARP6* and histone *H2A.Z* genes leads to reduced transcript levels of the genes within the thalianol and marneral clusters. (a) Analysis of thalianol and sitosterol levels in root extracts of *Arabidopsis thaliana* Col-0 wild-type and *arp6* mutant lines. Measurements were made relative to an internal standard, coprostanol, added before extraction. Extracts were analysed by GC-MS and peak areas of individual compounds were quantified (Supporting Information Fig. S2). Error bars indicate standard deviation of three biological replicates. Significant differences between wild-type and mutant lines (*t*-test): ***P <* *0.001. (b, c) Relative transcript levels of the thalianol cluster genes in the Col-0 wild-type and *arp6* mutant line (b) and *hta9/hta11* double mutant line (c). Black bars, thalianol pathway genes; grey bars, flanking genes. Transcript levels were measured by qRT-PCR as for Fig.1. Error bars indicate standard deviation of three biological replicates. Statistical significance (*t*-test): *P <* *0.05; **P <* *0.01. (d) H2A.Z abundance at thalianol cluster genes in chromatin preparations from roots and leaves of seedlings of *A. thaliana* accession Col-0. H2A.Z abundance was measured at the transcriptional start sites of the cluster genes and was quantified relative to *FLOWERING LOCUS C* (*FLC*). Error bars indicate standard deviation of three biological replicates. Significant differences in H2A.Z abundance between leaves and roots (*t*-test): *P <* *0.05; **P <* *0.01. (e, f) Relative transcript levels of the marneral cluster genes in the Col-0 wild-type and *arp6* mutant line (e) and *hta9/hta11* double mutant line (f). Black bars, marneral pathway genes; grey bars, flanking genes. Transcript levels were measured by qRT-PCR as for Fig.1. Error bars indicate standard deviation of three biological replicates. Statistical significance (*t*-test): *P <* *0.05; **P <* *0.01.

We next investigated the importance of ARP6 for the expression of metabolic gene clusters in *Arabidopsis thaliana* in detail. Thalianol levels were significantly reduced in root extracts of *arp6* mutant seedlings when compared with the wild-type, while levels of sitosterol (a representative sterol that is a product of primary metabolism and that has a common precursor with thalianol) were unaltered (Fig.2a, Supporting Information Fig. S2). Investigation of the transcript levels for all four thalianol cluster genes revealed that these were reduced in the *arp6* mutant background while those of the genes immediately flanking the cluster were not, indicating that ARP6 is required for normal cluster regulation (Fig.2b). These experiments were carried out in the Colombia (Col-0) accession of *A. thaliana*. Similar results were obtained with the *A. thaliana* Landsberg (L*er*) accession and its respective *arp6* mutant line (Martin-Trillo *et al*., 2006) (Supporting Information Fig. S3).

Since ARP6 is a SWR1 remodeller required for deposition of H2A.Z into nucleosomes and this histone variant has previously been shown to be important for regulation of the *DAL* gene cluster in yeast, we next investigated the role of H2A.Z in thalianol cluster regulation. To do this we used the *Arabidopsis thaliana* double mutant *hta9*/*hta11*, which is mutated in two of the three *A. thaliana* H2A.Z genes and has previously been shown by others to phenocopy the *arp6* mutant line (March-Diaz *et al*., 2008; Kumar & Wigge, 2010). The transcript levels of all four thalianol cluster genes were significantly reduced in the *hta9/hta11* mutant line compared with the wild-type, while those of the genes immediately flanking the clusters were unaffected (Fig.2c). The effects of the *arp6* and *hta9/hta11* mutations are therefore localized and specific to the cluster genes when compared with the flanking genes. These results implicate H2A.Z in ARP6-mediated cluster expression.

**Fig 3:**
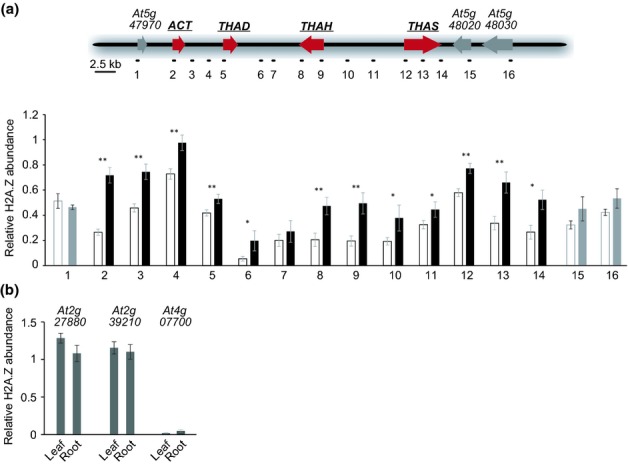
Chromatin immunoprecipitation (ChIP) analysis of H2A.Z abundance in the thalianol gene cluster. (a) H2A.Z abundance within the thalianol gene cluster was measured in chromatin preparations from leaves (white bars) and roots (black bars) of seedlings of *Arabidopsis thaliana* accession Col-0. H2A.Z abundance in different regions of the cluster was quantified relative to *FLOWERING LOCUS C* (*FLC*) (the white bars with grey outlines and the grey bars show data for regions outside the gene cluster). The probes used for this analysis (shown below the histogram) are indicated in the map of the thalianol cluster. Error bars indicate standard deviation of three biological replicates. Significance differences in H2A.Z abundance between leaves and roots (*t*-test): *P <* *0.05; **P <* *0.01. (b) H2A.Z abundance at selected control loci. *At2g27880* and *At2g39210* were used because both have been shown to be marked with the H2A.Z histone variant, have SWR1 dependent expression and do not show differential transcription rate between roots and leaves (based on AtGenExpress) (March-Diaz *et al*., 2008; Zilberman *et al*., 2008). The *At4g07700* locus was chosen as a negative control for the ChIP experiment, because it has been shown that H2A.Z is not incorporated into nucleosomes of this locus (Kumar & Wigge, 2010). H2A.Z abundance at these loci was quantified relative to *FLC*. Error bars indicate standard deviation of three biological replicates.

The role of H2A.Z in positive regulation of the thalianol cluster may be direct, or alternatively could be an indirect result of modifications elsewhere in the genome. We therefore analysed H2A.Z abundance in the thalianol gene cluster region to investigate whether H2A.Z is likely to directly regulate cluster expression. To do this we carried out ChIP experiments using an *HTA11:GFP* transgenic *Arabidopsis thaliana* line (Kumar & Wigge, 2010). H2A.Z deposition in *A. thaliana* is generally thought to be restricted to genic sequences, with peaks at the 5′ ends of target genes (Zilberman *et al*., 2008; Coleman-Derr & Zilberman, 2012). We initially chose the transcriptional start sites of the thalianol cluster genes as targets for our ChIP analysis. Comparison of H2A.Z abundance at these positions in chromatin isolated from roots and leaves revealed H2A.Z deposition in nucleosomes from both tissues, but with significantly higher levels in chromatin isolated from roots (the organ in which the thalianol cluster is expressed), suggesting a positive correlation between H2A.Z nucleosome incorporation and gene cluster transcription (Fig.2d). There were no significant organ-specific differences in H2A.Z levels in the genes immediately flanking the thalianol cluster. We next investigated whether the modifiable H2A.Z deposition was restricted to the genic regions within the cluster by measuring H2A.Z abundance using probes localized in different parts of the thalianol gene cluster, including the intergenic regions. We detected significantly higher H2A.Z levels throughout the whole cluster in roots when compared with leaves (Fig.3a). Thus H2A.Z deposition spans the entire cluster region and is not restricted to the individual cluster genes. By contrast, in nonclustered eukaryotic genes H2A.Z deposition is usually limited to gene bodies and the immediate vicinity of the transcriptional start site (Guillemette *et al*., 2005; Creyghton *et al*., 2008; Whittle *et al*., 2008; Coleman-Derr & Zilberman, 2012). As controls, we analysed H2A.Z levels at three loci previously reported to show either presence (*At2g27880* and *At2g39210*) or absence (*At4g07700*) of H2A.Z (March-Diaz *et al*., 2008; Kumar & Wigge, 2010). We detected constant H2A.Z deposition at *At2g27880* and *At2g39210* and precipitated only minor amounts of DNA at the transcriptionally silent locus *At4g07700*, as expected (Fig.3b).

Similar results were obtained for the marneral cluster (Fig.2e,f, Supporting Information Fig. S4). For both the thalianol and marneral clusters, the effects of the *arp6* and *hta9/hta11* mutations and the incorporation of H2A.Z were localized and specific to the clusters when compared with the genes immediately flanking the clusters. The transcript levels of the thalianol and marneral cluster genes were not completely abolished in *arp6* mutants, indicating that additional as yet unidentified regulatory components also contribute to gene cluster expression. However our results provide evidence for a role for ARP6 and H2A.Z in positive regulation of the thalianol and marneral clusters through localized chromatin remodelling.

**Fig 4:**
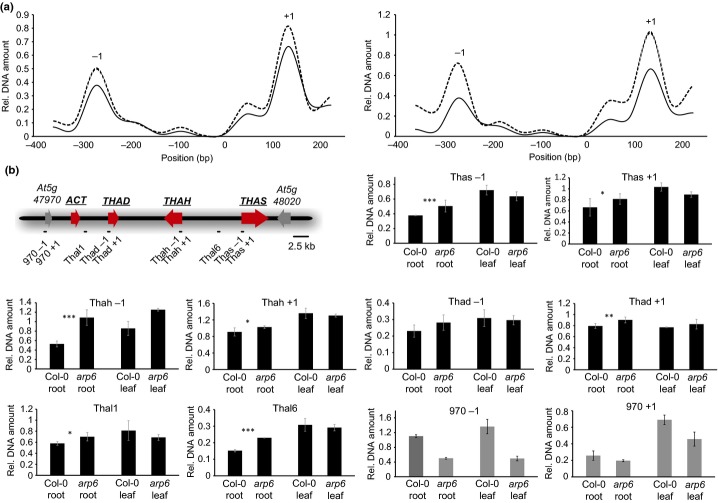
Analysis of nucleosome occupancy within the thalianol cluster. (a) Comparison of nucleosome positioning at the transcriptional start site of the *THAS* gene in roots of *Arabidopsis thaliana* Col-0 wild-type (solid line) and *arp6* mutant (dashed line) lines (left); Col-0 roots (solid line) and leaves (dashed line) (right).The −1 and +1 indicate the first positioned nucleosome upstream and downstream of the putative transcriptional start site. (b) Relative abundance of stably positioned nucleosomes within the thalianol cluster and at the *At5g47970* flanking gene in roots and leaves of seedlings of Col-0 wild-type and *arp6* mutant lines. The position of each analysed nucleosome is indicated in the map of the cluster region. The −1 and +1 indicate the first positioned nucleosome upstream and downstream of the putative transcriptional start site of the respective gene. Nucleosome abundance was measured by qRT-PCR relative to the stably positioned −1 nucleosome at the *At4g07700* reference locus. Results for representative experiments are shown (error bars indicate standard deviation of two biological replicates). Significant differences between wild-type and mutants lines (five experiments, 10 biological replicates) (*t*-test): *P <* *0.05; **P <* *0.01; ***P <* *0.001.

We next analysed the local genome accessibility at the thalianol cluster using micrococcal nuclease accessibility assays. Addition of micrococcal nuclease to nuclear chromatin leads to digestion of nucleosome-free DNA, while nucleosome-bound DNA is protected. Subsequent quantification of undigested DNA enables the identification of nucleosome positions and their stability at these sites. Initially, we compared the nucleosome abundance at the transcriptional start-site of the *THAS* gene within the thalianol gene cluster. As expected we found two well-positioned nucleosomes, the **−**1 nucleosome, just upstream of the transcriptional start site and the +1 nucleosome, just downstream (Fig.4a). In preliminary experiments we found that the −1 and +1 nucleosomes were less abundant in chromatin preparations from wild-type roots compared with the transcriptionally less active *arp6* roots, or to wild-type leaves (where the cluster is inactive) (Fig.4a). We then broadened our analysis to the whole cluster (Fig.4b). We detected well-positioned nucleosomes around the putative transcriptional start sites of thalianol cluster genes and elsewhere within the gene cluster, and measured the abundance of these nucleosomes in chromatin isolated from the roots and leaves of wild-type and *arp6* mutant *Arabidopsis thaliana* lines. In seven out of the eight positioned nucleosomes analysed we found a significant increase in nucleosome occupancy in the *arp6* mutant roots compared with the wild-type (Fig.4b). Surprisingly, we found higher nucleosome occupancy not only at the transcriptional start sites of cluster genes but also at intergenic regions within the cluster, in line with the cluster-wide enrichment of H2A.Z.

H2A.Z deposition may be involved in either opening or compaction of chromatin environments in *Arabidopsis thaliana* (Deal *et al*., 2007; Kumar & Wigge, 2010). Generally, higher nucleosome density is correlated with a less accessible chromatin structure, which hinders the ability of the transcriptional machinery to associate with its target genes. Our data suggest that ARP6-mediated incorporation of H2A.Z into nucleosomes within the thalianol and marneral gene clusters may lead to localized opening of the chromatin structure within the cluster regions, so facilitating cluster expression. The nucleosomes analysed at the *At5g47970* gene, located directly upstream of the thalianol gene cluster, did not show an increase in nucleosome abundance in the *arp6* mutant background compared with the wild-type. We also observed more stably positioned nucleosomes in leaf tissues compared with root tissues, consistent with the correlation between transcriptional activity of the gene cluster and more loosely organized nucleosomes. This is in accordance with our previous DNA fluorescence *in situ* hybridization experiments, which suggested that the avenacin metabolic gene cluster in oat switches between a highly condensed and an open chromatin structure during transition from the repressed to the actively transcribed state (Wegel *et al*., 2009). We also found a similar decrease in root stable nucleosome positioning at one of the thalianol cluster flanking genes (the *At5g47970* gene), which does not show differential regulation in root and leaf tissues. We suggest that the more open nucleosome structure of the highly expressed thalianol gene cluster spreads into this neighbouring region independently of H2A.Z.

The rapidly growing number of reports of metabolic gene clusters for the synthesis of diverse classes of compound from different plant species suggests that this form of genomic organization is common in plants, although a comprehensive overview of the overall relative frequencies of clustered and dispersed pathways for specialized metabolism in plant genomes is not yet known. The physical clustering of genes whose functions are related but that do not share sequence similarity and have not arisen by tandem duplication is intriguing. Clustering should favour co-inheritance of beneficial combinations of alleles that together confer a selective advantage. As we show here, it is also likely to be important for coordinate regulation of expression of pathway enzymes. The synthesis of specialized metabolites in plants is normally tightly regulated. There is evidence for the thalianol and marneral clusters and also for some other plant metabolic clusters that the pathway intermediates are toxic, and so strict regulation may be critical in avoiding build-up of these toxic compounds (Field & Osbourn, 2008; Mylona *et al*., 2008; Field *et al*., 2011; Takos *et al*., 2011). Our data support the hypothesis that clustering facilitates pathway regulation, implicating the SWR1 remodelling complex component ARP6 and the histone 2 variant H2A.Z in the regulation of discrete metabolic gene clusters of *Arabidopsis thaliana* through highly localized chromatin modifications that enable the coordinate expression of windows of contiguous genes. A possible scenario is that incorporation of H2A.Z into nucleosomes at the thalianol and marneral loci may poise these clusters for rapid expression in an otherwise highly repressive heterochromatin region, so making the genes within them accessible to transcription factors, as has been shown to be the case for the *DAL* gene cluster for allantoin catabolism in yeast (Meneghini *et al*., 2003; Guillemette *et al*., 2005; Wong & Wolfe, 2005). Alternatively ARP6 and H2A.Z may have other indirect roles in cluster regulation through as yet undefined mechanisms. While mutations that affect ARP6-mediated H2A.Z deposition have a clear effect on cluster expression and metabolite production they do not abolish cluster activity completely, indicating that other factors are also likely to be involved. Chromatin regulation of specialized metabolism is not limited to clustered pathways, and several previous studies have shown that mutations in important chromatin regulators can affect the synthesis of compounds such as phenylpropanoids and glucosinolates, pathways for which the genes not regarded as clustered in *A. thaliana* (Rider *et al*., 2004; Bennett *et al*., 2005; Walley *et al*., 2008). It is not clear, however, whether these effects are a direct consequence of chromatin remodelling at the cognate biosynthetic loci or a secondary effect of modifications elsewhere in the genome. Future work will compare mechanisms of regulation between clustered and nonclustered pathways and investigate the roles of other regulatory processes in cluster regulation, and is expected to open up new opportunities for the discovery and manipulation of specialized metabolic pathways in plants.

## Supporting Information

**Fig. S1**Relative transcript level of the thalianol and marneral cluster genes in RNA preparations from leaf and root tissue of the Col-0 wild-type. **Fig. S2** GC-MS analysis of Col-0 and *arp6* root extracts. **Fig. S3** Relative transcript levels of the thalianol and marneral cluster genes in L*er* wild-type and corresponding *arp6* mutant line. **Fig. S4** Chromatin immunoprecipitation (ChIP) analysis of H2A.Z abundance at the marneral gene cluster and control loci. **Table S1***Arabidopsis thaliana* mutant lines **Table S2** Oligonucleotides used in this study
