# Supplementary material for: Regulation of metabolic gene clusters in Arabidopsis thaliana
Source: New Phytol. 2014 Nov 21;205(2):503–10. doi: 10.1111/nph.13189 (PMC4301183; doi:10.1111/nph.13189)
Supplement: Supplementary file 1 — Fig. S1Relative transcript level of the thalianol and marneral cluster genes in RNA preparations from leaf and root tissue of the Col-0 wild-type. Fig. S2 GC-MS analysis of Col-0 and arp6 root extracts. Fig. S3 Relative transcript levels of the thalianol and marneral cluster genes in Ler wild-type and corresponding arp6 mutant line. Fig. S4 Chromatin immunoprecipitation (ChIP) analysis of H2A.Z abundance at the marneral gene cluster and control loci. Table S1Arabidopsis thaliana mutant lines Table S2 Oligonucleotides used in this study [file nph0205-0503-sd1.pdf]

New Phytologist **Supporting Information**

Article title: **Regulation of metabolic gene clusters in *Arabidopsis thaliana***

Authors: Hans-Wilhelm Nützmann and Anne Osbourn

Article acceptance date: 23 October 2014

The following Supporting Information is available for this article:

Fig. S1 Relative transcript level of the thalianol and marneral cluster genes in RNA preparations from leaf and root tissue of the Col-0 wild type.
Fig. S2 GC-MS analysis of Col-0 and *arp6* root extracts.
Fig. S3 Relative transcript levels of the thalianol and marneral cluster genes in L*er* wild type and corresponding *arp6* mutant line.
Fig. S4 ChIP analysis of H2A.Z abundance at the marneral gene cluster and control loci.

Tables S1 *A. thaliana* mutant lines
Tables S2 Oligonucleotides

Fig. S1 Relative transcript level of the thalianol and marneral cluster genes in RNA preparations from leaf and root tissue of the Col-0 wild type.

(**a**) Thalianol gene cluster

(**b**) Marneral gene cluster

Black bars indicate cluster genes and grey bars indicate flanking genes. Gene expression level was measured by qRT-PCR and leaf transcript level rate was set as 1. *PP2AA3* (*At1g13320*) was used as internal control (Hong *et al.*, 2010). Error bars indicate standard deviation of three biological replicates.

(**a**)

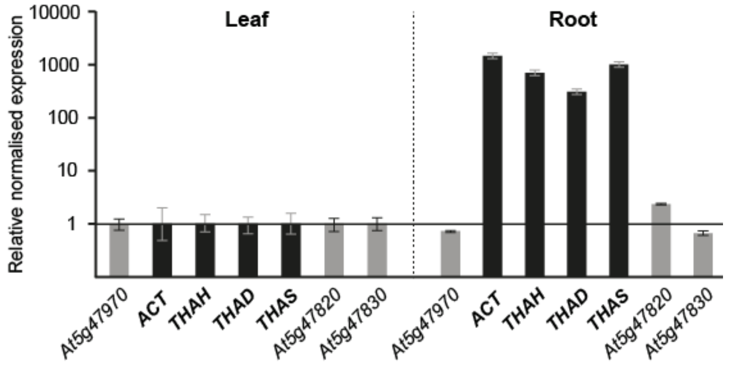

(**b**)

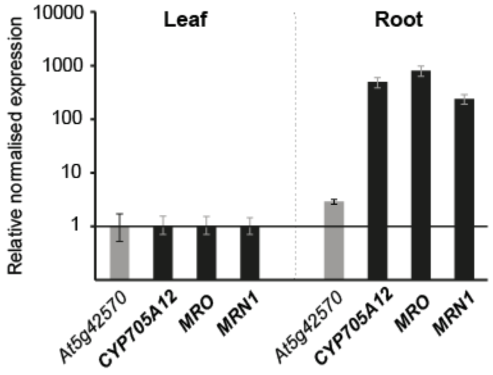

Fig. S2 GC-MS analysis of Col-0 and *arp6* root extracts.

Extracted ion chromatograms (*m/z* 229) for Col-0 and *arp6* root extracts are shown. The thalianol, coprostanol and sitosterol peaks are indicated.

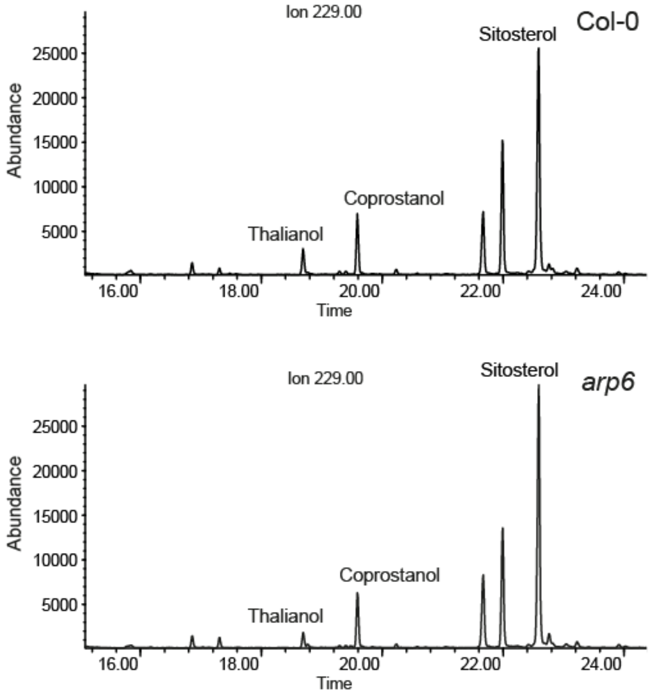

Fig. S3 Relative transcript levels of the thalianol and marneral cluster genes in L*er* wild type and corresponding *arp6* mutant line.

Transcript levels were measured by qRT-PCR and wild type transcript rate was set as 1. *PP2AA3* (*At1g13320*) was used as internal control (Hong *et al.*, 2010). Error bars indicate standard deviation of three biological replicates.

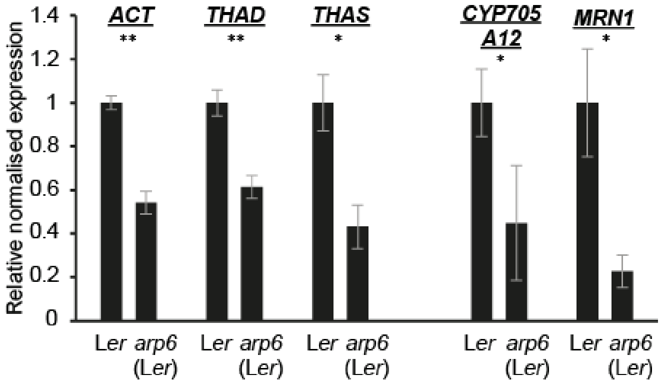

Fig. S4 ChIP analysis of H2A.Z abundance at the marneral gene cluster and control loci.

H2A.Z abundance within the marneral gene cluster was measured in chromatin preparations from root and leaf tissue of *A. thaliana* by ChIP coupled to qRT-PCR. The cluster is transcribed in the roots and silent in the leaves. H2A.Z abundance was quantified relative to *FLC*. The probes used for this analysis are indicated in the map of the marneral cluster. Error bars indicate standard deviation of three biological replicates. Asterisks indicate statistical significance (*t*-test; *, *P*<0.05;**, *P*<0.01).

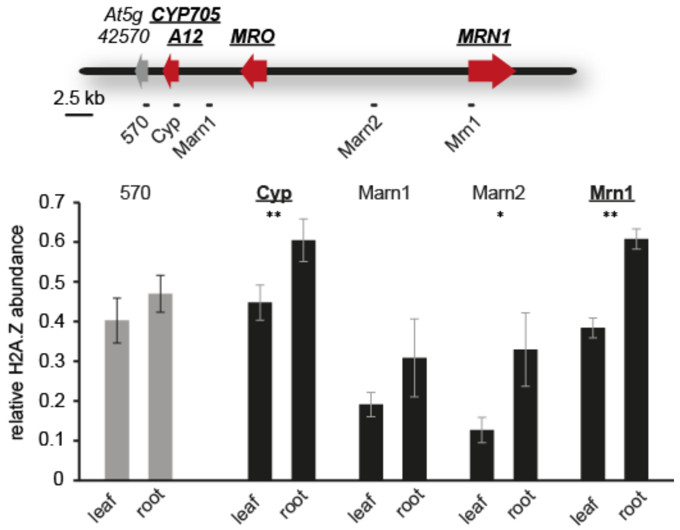

Table S1 *A. thaliana* mutant lines

The *A. thaliana* mutant lines were chosen based on described chromatin regulatory function of the disrupted genes. Special emphasis was given to lines that are affected in their histone modifications and structure.

**Line**	**Gene function**	**Reference**
atxr7	histone methyltransferase	Tamada *et al.*, 2009
suvh2	histone methyltransferase	Naumann *et al.*, 2005
atx1	histone methyltransferase	Pien *et al.*, 2008
ref6	histone demethylase	Noh *et al.*, 2004; Lu *et al.*, 2011
atjmj4	histone demethylase	Noh *et al.*, 2004
elf6	histone demethylase	Noh *et al.*, 2004; Jeong *et al.*, 2009
hda6	histone deacetylase	Murfett *et al.*, 2001; Probst *et al.*, 2004
vim1	binds to methylated DNA	Woo *et al.*, 2007
prmt5	histone arginine methyltransferase	Pei *et al.*, 2007
prmt10	histone arginine methyltransferase	Niu *et al.*, 2007
otld1	histone deubiquitinase	Krichevsky *et al.*, 2011
vin3-8	subunit of the polycomb response complex	Sung & Amasino, 2004; Bond *et al.*, 2009
arp6 (Col-0)	subunit of SWR1 complex	Deal *et al.*, 2007
haf1	histone acetyltransferase	Crane & Gelvin, 2007
hda18	histone deacetylase	Xu *et al.*, 2005

Table S2 Oligonucleotides

qRT-PCR

Oligo	Forward	Reverse
AT1G13320	GTTGTGGAGAACATGATACGG	GCTAGACATCATCACATTGTC
AT5G42570	GGTTTCTTATGGAGTATGATAGGTT	TTCTTTCCCTCAGGACT
CYP705A12	CCACAAACTACTCCGACCAC	CTTCTTCTTCGCCTTACCTTG
MRO	ATCACGAAATCTGCCCATAAA	CTTCCAGCGTCTCCATCAATA
MRN	ATGCTTGCTTGTTGGGTAGAA	TTTGCGGCTAACATAACTTGG
AT5G47970	GGATACCAACGAGACCTGTG	CATCACATCTTTGTAGCTGCATG
ACT	CGGCCATACTAACACTACACT	TGCCTACCCATACTTCAACAG
THAD	ATCCTGATTTCTGGGAAGACC	TGTTGCACCATCATTCCAATT
THAH	TGGTGTTTGGAGGTGGAGTGA	GGGAAATCTTGATAGGCAGTC
THAS	CTTCAATCCACTATGGCACTC	TAAAATAATCACTCTTAGGGTCTT C
AT5G48020	TTACAACGAGGAAGGGACGAT	ACCGGAACTTTAACCGCAACC
AT5G48030	GACAGCGTGAACTGCTTGAG	CACTGGGAAGATCCAGTTGC

ChIP

Oligo	Forward	Reverse
AT5G42570	GACCGACACAACAAACAACAC	GAAGGATCAGTGCCATTTCG
AT5G42580	GCAGAACTCATCATCGTTGAC	GACCGATGATTGGTAGAGAAG
MRO	CGATACACCCAAAATCAAATC	GTTAGGAAAGTGGTTAAGCAC
MRN	CACGTTAACCAGTTAGCAGATTG	GTTGGCATCAAACTCCCAGATC
AT5G47970	GAGGCGATAGAAATGACTTAGTTG	GACCCACTTTCGTTGGTGTTC
ACT	TCAAGCTTAACCAATATCACGTG	CGATGACTGATAGTTGAAGGC
THAD	GAAAACTGCTTCATCTTCCTCC	GATTGGTAATGAAGGAGGGCTC
THAH	TAAGAGAAGTGTACTTGATATTCGG	CGACTGCTTAACCTACCTTTG
THAS	GTCCAAGTAAACTTAGAGCAACC	CTGCGTTGGCATCAAATTCC

AT5G48020	CAATGATGAATGAACAATGGAG	CAGTAGTGACCAGTTCTGTAG
AT5G48030	CTAGGGTTTTAACTTTGGCTGC	CCATTGGAAGGGACCATTTTC
Thal1	GATGAAGAGTGGCATGAGGC	CGAAAGGCATGTCTTCGCTC
Thal2	GTTATCCGACTCATGCAATGC	GCATAGTGAAGACAGAGAGTTG
Thal3	CATGATTACCTGTTAGAGGAG	GTCAACGCAACAGAGAATTTC
Thal4	CAACGTGATTTTATTCTTATGG	GTATTGAAAACACCCGTTAAG
Thah2	GAAGTATTTTCAATGGGGCAATAC	GTTGGAAGTTTCATCTTTGAATAC C
Thal5	GATACCTAGAAGGATTCCACG	CTTTTGCCCTTCCAACCTATAG
Thal6	CATCTTTTGGCAAGTTTCACTCTC	GATTCATGAAGCACATGGTCG
Thas2	CACATGTTCTCAGAGAGTATC	CACAACGACTACCTTTGGCAG
Thas3	CCTGGATAAGAGAACAAGTGC	CTTGTACTCTTTGAACACATTCAAC

Nucleosome Positioning

Oligo	Forward	Reverse
THASnupo1	GATGACAATGCGAAACGTAGTTG	CACGATGCATGTGAGAAACGG
THASnupo2	CGTTAAGGAATAAAATTATTTG	CCACAAAAATACGTAGAAAAG
**THASnupo3**	**CACACCCGTTTCTCACATGC**	**CATCAACCACAAAAATACGTAG**
THASnupo4	CTTTTCTACGTATTTTTGTGG	AACAATGTGTTAGTTTTGAG
THASnupo5	GGTTGATGTTTAAAATTTTTAC	TGGTTTGTTTGAGTTATAATAG
THASnupo6	CTCAAAACTAACACATTGTT	GTTTTCAAATTTTAATACAAACC
THASnupo7	CTATTATAACTCAAACAAACCA	CTTGCAATTAAATTATGTTTTCA
THASnupo8	CATTTACATAATAGAAATCATC	CGCTTCGTATTTTTTATAGTCG
THASnupo9	GGTTTGTATTAAAATTTGAAAAC	GGACAAGGAGGACACATTAT
THASnupo10	CCTAAAACATCGACTATAAAA	CAGCCTCCACATTTTGTAAGG
THASnupo11	GTATAATGTGTCCTCCTTGTCC	GTTGGTGGTGAACAGGTGAG
**THASnupo12**	**CCTTACAAAATGTGGAGGCTG**	**GCATCAAATTCCCAAATCTGC**
THASnupo13	GGAGAGGATACTCACCTGTTC	GAATTTGTGCCGAGCATCCTC

THASnupo14	CTATGCAGGGAGGCAGATTTG	GTAGTCTTGAAACGTGACGTG
THASnupo15	CACAAGAAATTGCCGAGGTAG	CAAAGTATTTCACCTGCATGC
47970nupo1	CTGAACTCGTTTCTTGAAAACC	GCAATTTCAATCAGAAGATAG
47970nupo2	CGATCAAGACTACCTTGAACAC	CCTCTAAAATCTATAAGCTTGTG
47970nupo3	CTGAAATTTTGACATCGTTCAC	CTCCTCTTCTTATTACATCAGAG
47970nupo4	GAGGCGATAGAAATGACTTAGTTG	GATGCTTCACAAGTCGCAAC
**47970nupo5**	**GAGGAGAGATCTAAAAGATGAAAG**	**GACCCACTTTCGTTGGTGTTC**
47970nupo6	CTTGTGAAGCATCGGGAGAC	GAATGACGTCAAAAAGGAATG
47970nupo7	GTCCCGCCTTTTGTTTCCTT	GACGAGAGAGGGTGAAATTGG
47970nupo8	GCAAATCTCAGCGACCACAA	GACGAATACAGGCAGCTATAG
**47970nupo9**	**GTGTTCTTGTCTGAAACTCG**	**GTTGGTAAATCTCAGCTTCTC**
47970nupo10	GACCAGCAGTGTCCCATATC	CAGTATCGAATCTAAAGCAACG
47970nupo11	CTAAAACAAGGATCTACGATGG	CAAGAACACTTGAAATCGATCG
47970nupo12	GCGACTGCCAAGCGAAATTC	GACACTGCTGGTCAAGAACG
THADnupo1	CATTGATCAATGCATGGGATC	GATCATTGATGCCATTGTTGC
THADnupo2	GGCTACTCTGTGTCATTTGC	CATAAGAGGAGGAAGATGAAG
THADnupo3	CAATGGCATCAATGATCACTG	CTGAAGAAAAGATCGTAAGAG
**THADnupo4**	**CATCTTCCTCCTCTTATGTC**	**GAGCACAGCCAGCTCGTGAG**
THADnupo5	CTTACGATCTTTTCTTCAGG	GGTGAAGATGACCAATGATTG
**THADnupo6**	**GAGAAAACATATTATATTTAGG**	**GCAAATGACACAGAGTAGCC**
THADnupo7	CTTAAAAAGTAATGACGTCAGC	CATGCATTGATCAATGATCAG
THADnupo8	GTGCTTGTCCCAGAGAACAAC	GATCAGGCTTTGTTTATTACC
THADnupo9	CCAATGTGAATGCTATAACG	GAAAGCTGACGTCATTACT
THADnupo10	GACACTCTTTATTTTATTATC	CTGGGACAAGCACAATCGAG
THADnupo11	CAGATGTTTAAAATGTCTCT	CATTGGTTAATTTTTGAAGG
THADnupo12	GGTGTCTTTAGTATAAATTAG	GATAATAAAATAAAGAGTGTC
THADnupo13	CCAATAAGTCCCAACATCCAC	AGAGACATTTTAAACATCTG

THADnupo14	GTAAGTTGAAACCTCTATAGC	CTAATTTATACTAAAGACACC
THADnupo15	GACATTCACAACTTCTACAG	GGACTTATTGGTTTGAGTGG
THADnupo16	CAATTTTAACATAATTTATATTC	GATTAGTAACTAAAAACTGTAG
THADnupo17	TTGTTACTATGTTGCAAC	CTGTAGAAGTTGTGAATGTC
THAL6nupo2	GAATAGGAATATGAAAAAAGTG	GATAGGTAGAGATGATCATACC
THAL6nupo3	CACAGTAGAATCCATACTCAC	GTGAAACTTGCCAAAAGATG
THAL6nupo4	CGTTGCAACTTACAACCATAC	CCTATTCACCTTACGATGATG
**THAL6nupo5**	**GACTAGAAAAAACGTGGGACC**	**GACTTCTAAGAGGTAAAGAGG**
THAL6nupo6	CAAATATTCAAGTTTCCCGGAC	GTTGTAAGTTGCAACGCCATG
THAL6nupo7	GCAATCACTTGAGTGAAAAC	GTCTTTCGATGATTAGTACG
THAL6nupo8	CAATTAGTGATGCTTGAGCTAG	CCACAATATTTTTATGTTTTG
THAL1nupo1	CTTTCAAAGATGCATCTGTTGC	GACGCAACTGATTTTTTGTAGC
THAL1nupo2	GAGATGAAGAGTGGCATGAGG	CAGAGTTAGCTAATAATATGGC
THAL1nupo3	GCTACAAAAAATCAGTTGCGTC	GCTCAGATCTTTCCAATGTG
THAL1nupo4	CTAACTCTGAAGTTACAAATCC	GTTAACTTCCTATGCTCGAAAG
THAL1nupo5	CACATTGGAAAGATCTGAGCG	CTCCATTACACTATAACACTGC
THAL1nupo6	GGAAGTTAACTAATTTAAATG	GTTGGAATAATCAACAAATGAC
THAL1nupo7	GTGTAATGGAGTTCGAGTC	CTTGGTTGATAAACTGTAGC
**THAL1nupo8**	**GTTGATTATTCCAACATGCC**	**CTTCATGCAATCGAACTTTATC**
THAL1nupo9	CCAAGTTATATAACCCAATG	CATCGTTTATGTTCTCCACTTC
THAHnupo1	CAAAAGGTGTTTACAACCTTC	CCTCTTCATTTTCAAAATGT
THAHnupo2	TTGAAACTATATAGTATCATG	CTTGGTAAGTACTGGAACCTC
THAHnupo3	ACATTTTGAAAATGAAGAGG	CACCCCACTTTTGACAAATC
THAHnupo4	GTACTTACCAAGTTTTAAAAC	GAAAAGTTGTGTCTCACAAG
THAHnupo5	GATTTGTCAAAAGTGGGGTGC	GGTTAATATATTTCTTCAGG
THAHnupo6	GAGACACAACTTTTCTTCATAG	CAAGTACACTTCTCTTATAACC
THAHnupo7	TGAGTAACACTATCCTGAAG	CAAATTATACCTAGTTTTTGG

THAHnupo8	GTATAGGTTATAAGAGAAGTG	CACCTCAACTTAGACCTAG
THAHnupo9	CCAAAAACTAGGTATAATTTG	CAAATAACTTTATACTAGACC
THAHnupo10	CTAGGTCTAAGTTGAGGTG	CAACGACTGCTTAACCTACC
THAHnupo11	GGTCTAGTATAAAGTTATTTG	GTCATTACAAACAAACAACTC
THAHnupo12	CCAAAGGTAGGTTAAGCAGTC	GAATAATCTTAACCTACCTATG
THAHnupo13	GTCTGCACTCTGCAAAAAAC	TAGTTTCATGAAGAAGGTTG
THAHnupo14	GTATACTATACATGTTAACG	GTAAACACCTTTTGTAATTGC
THAHnupo15	GTGAGCTTCTAACCTAAAATC	GATAATATATGTTTTTTGCAG
THAHnupo16	GGTTGAGTATGTGCGGATCG	CGTTAACATGTATAGTATAC
THAHnupo17	GTTATATTGAGTTGTTTGTTTG	GACCACACGAAGCTCATTTG
THAHnupo18	CATAGGTAGGTTAAGATTATTC	CAACAGCGGCTACAGCTATG
**THAHnupo19**	**CAAATGAGCTTCGTGTGGTC**	**CATTGCACTTCGGGTTCGAC**
**THAHnupo20**	**GTTTGGGCATGGATCGACG**	**CTCTAATATGGTATCAGAGCC**
07700nupo -1	CACATTCCTCAGCCGTTGATG	CTATCTCCCAGCTAGCAACC

Bold, oligos used for stably positioned nucleosomes in thalianol gene cluster.

References

Bond DM, Wilson IW, Dennis ES, Pogson BJ, Finnegan EJ. 2009. *VERNALIZATION INSENSITIVE 3* (*VIN3*) is required for the response of *Arabidopsis thaliana* seedlings exposed to low oxygen conditions. *Plant Journal* **59**(4): 576-587.

Crane YM, Gelvin SB. 2007. RNAi-mediated gene silencing reveals involvement of *Arabidopsis* chromatin-related genes in Agrobacterium-mediated root transformation. *Proceedings of the National Academy of Sciences, USA* **104**(38): 15156-15161.

Deal RB, Topp CN, McKinney EC, Meagher RB. 2007. Repression of flowering in *Arabidopsis* requires activation of *FLOWERING LOCUS C* expression by the histone variant H2A.Z. *Plant Cell* **19**(1): 74-83.

Hong SM, Bahn SC, Lyu A, Jung HS, Ahn JH. 2010. Identification and testing of superior reference genes for a starting pool of transcript normalization in *Arabidopsis*. *Plant Cell Physiology* **51**(10): 1694-1706.

Jeong JH, Song HR, Ko JH, Jeong YM, Kwon YE, Seol JH, Amasino RM, Noh B, Noh YS. 2009. Repression of *FLOWERING LOCUS T* chromatin by functionally redundant histone H3 lysine 4 demethylases in *Arabidopsis*. *PLoS One* **4**(11): e8033.

Krichevsky A, Zaltsman A, Lacroix B, Citovsky V. 2011. Involvement of KDM1C histone demethylase-OTLD1 otubain-like histone deubiquitinase complexes in plant gene repression. *Proceedings of the National Academy of Sciences, USA* **108**(27): 11157-11162.

Lu FL, Cui X, Zhang SB, Jenuwein T, Cao XF. 2011. *Arabidopsis* REF6 is a histone H3 lysine 27 demethylase. *Nature Genetics* **43**(7): 715-U144.

Murfett J, Wang XJ, Hagen G, Guilfoyle TJ. 2001. Identification of *Arabidopsis* histone deacetylase HDA6 mutants that affect transgene expression. *Plant Cell* **13**(5): 1047-1061.

Naumann K, Fischer A, Hofmann I, Krauss V, Phalke S, Irmler K, Hause G, Aurich AC, Dorn R, Jenuwein T *et al*. 2005. Pivotal role of AtSUVH2 in heterochromatic histone methylation and gene silencing in *Arabidopsis*. *EMBO J* **24**(7): 1418-1429.

Niu LF, Lu FL, Pei YX, Liu CY, Cao XF. 2007. Regulation of flowering time by the protein arginine methyltransferase AtPRMT10. *EMBO R.* **8**(12): 1190-1195.

Noh B, Lee SH, Kim HJ, Yi G, Shin EA, Lee M, Jung KJ, Doyle MR, Amasino RM, Noh YS. 2004. Divergent roles of a pair of homologous jumonji/zinc-finger-class

transcription factor proteins in the regulation of *Arabidopsis* flowering time. *Plant Cell* **16**(10): 2601-2613.

Pei YX, Niu LF, Lu FL, Liu CY, Zhai JX, Kong XF, Cao XF. 2007. Mutations in the type II protein arginine methyltransferase AtPRMT5 result in pleiotropic developmental defects in *Arabidopsis*. *Plant Physiology* **144**(4): 1913-1923.

Pien S, Fleury D, Mylne JS, Crevillen P, Inze D, Avramova Z, Dean C, Grossniklaus U. 2008. ARABIDOPSIS TRITHORAX1 dynamically regulates *FLOWERING LOCUS C* activation via histone 3 lysine 4 trimethylation. *Plant Cell* **20**(3): 580-588.

Probst AV, Fagard M, Proux F, Mourrain P, Boutet S, Earley K, Lawrence RJ, Pikaard CS, Murfett J, Furner I *et al*. 2004. *Arabidopsis* histone deacetylase HDA6 is required for maintenance of transcriptional gene silencing and determines nuclear organization of rDNA repeats. *Plant Cell* **16**(4): 1021-1034.

Sung SB, Amasino RM. 2004. Vernalization in *Arabidopsis thaliana* is mediated by the PHD finger protein VIN3. *Nature* **427**(6970): 159-164.

Tamada Y, Yun JY, Woo SC, Amasino RM. 2009. *ARABIDOPSIS TRITHORAX-RELATED7* is required for methylation of lysine 4 of histone H3 and for transcriptional activation of *FLOWERING LOCUS C*. *Plant Cell* **21**(10): 3257-3269.

Woo HR, Pontes O, Pikaard CS, Richards EJ. 2007. VIM1, a methylcytosine-binding protein required for centromeric heterochromatinization. *Genes & Development* **21**(3): 267-277.

Xu CR, Liu C, Wang YL, Li LC, Chen WQ, Xu ZH, Bai SN. 2005. Histone acetylation affects expression of cellular patterning genes in the *Arabidopsis* root epidermis. *Proceedings of the National Academy of Sciences, USA* **102**(40): 14469-14474.
